# Google Search as an Additional Source in Systematic Reviews

**DOI:** 10.1007/s11948-017-0010-4

**Published:** 2017-12-16

**Authors:** Jan Piasecki, Marcin Waligora, Vilius Dranseika

**Affiliations:** 1 REMEDY, Research Ethics in Medicine Study Group, Department of Philosophy and Bioethics, Faculty of Health Science, Jagiellonian University Medical College, Kraków, Poland; 2 Department of Logic and History of Philosophy, Faculty of Philosophy, Vilnius University, Vilnius, Lithuania

Marko Curkovic in his letter (2017) points out that using Google Search in our systematic review (Piasecki et al. 2017) could have led to the so-called “bubble effect”, a form of selection bias. We take seriously this concern. Google Search is indeed an imperfect tool to perform systematic reviews: the search algorithm is not known and cannot be controlled, Google adapts the search to each user in order to personalize information and, as a result, a systematic search is quite probably not replicable. To avoid problems related to a personalized search, the primary source of the data in our study was a systematic search in PubMed. Searches in Google Scholar and Google Search were considered to be additional sources only. We expected that the total number of documents will be low, and we were rather more concerned with the comprehensiveness of our search than its representativeness, having in mind the qualitative, not the statistical character of the study. Moreover, in order to avoid personalization of search results, we logged off from all Google accounts. We regret that we did not describe this step in our paper. As a result, Google Search allowed to identify three additional guidelines.

Google Scholar and Google Search are considered to be important sources of grey literature, governmental and institutional reports (Haddaway et al. 2015; Hagstrom et al. 2015). In performing our study, we assumed that not all the guidelines have been published in scientific journals. Therefore, although Google Scholar and Google Search have their limitations and should not be used as the only source for systematic reviews, both seemed to be apt for the purposes of some types of qualitative systematic reviews.
